# Roles of Fibroblast Growth Factors in the Axon Guidance

**DOI:** 10.3390/ijms241210292

**Published:** 2023-06-18

**Authors:** Weiyun Zhang, Peiyi Luo, Xiaohan Liu, Ruoxi Cheng, Shuxian Zhang, Xiao Qian, Fang Liu

**Affiliations:** 1 Queen Mary School, Medical College, Nanchang University, Nanchang 330000, Chinajp4217118229@qmul.ac.uk (X.Q.); 2 Medical Experimental Teaching Center, School of Basic Medical Sciences, Nanchang University, Nanchang 330031, China; 3 Department of General Surgery, Second Affiliated Hospital of Nanchang University, Nanchang 330006, China; 4 Department of Cell Biology, School of Basic Medical Sciences, Nanchang University, Nanchang 330031, China

**Keywords:** fibroblast growth factors (FGFs), FGF receptors (FGFRs), axon guidance, nervous system

## Abstract

Fibroblast growth factors (FGFs) have been widely studied by virtue of their ability to regulate many essential cellular activities, including proliferation, survival, migration, differentiation and metabolism. Recently, these molecules have emerged as the key components in forming the intricate connections within the nervous system. FGF and FGF receptor (FGFR) signaling pathways play important roles in axon guidance as axons navigate toward their synaptic targets. This review offers a current account of axonal navigation functions performed by FGFs, which operate as chemoattractants and/or chemorepellents in different circumstances. Meanwhile, detailed mechanisms behind the axon guidance process are elaborated, which are related to intracellular signaling integration and cytoskeleton dynamics.

## 1. Introduction

The central nervous system consists of billions of neurons in humans, and the correct wiring of these neurons is essential for the establishment of the highly ordered cellular organization of the nervous system. Many molecular cues play important roles in ensuring the integrity and precision of the wiring of neural circuitry [1,2,3]. Extensive research has illustrated the biological functions of many classical guiding molecules, including netrins, slits, semaphorins and ephrins, as well as nerve growth factor (NGF) and brain-derived neurotrophic factor (GDNF) [1,4,5]. Recently, mounting pieces of evidence have described that fibroblast growth factors (FGFs) can function as guidance cues to control the pathway finding of distinct kinds of axons, exactly as other conventional morphogens such as Sonic hedgehog (Shh), bone morphogenetic protein (BMP) and Wnt factors [6,7,8,9].

The FGFs comprise a sizable family of secreted polypeptides that communicate with FGF receptors (FGFRs) to control various developmental processes, such as cell division, proliferation, differentiation, survival and migration, etc. [10]. Recent genetic modification experiments have improved our understanding of the biological importance of FGFs and FGFRs [10,11]. It has been demonstrated that several FGF subfamily members have an impact on the development of the central nervous system [12]. However, due to the FGFs’ overlapping expression patterns, they compensate for each other, so many of their precise activities may go unidentified. Moreover, interactions between FGFs and several other signaling molecules surely complicate the study procedure. Therefore, to identify and characterize the specific mechanisms and functions of different FGFs on various axons, in vivo and in vitro studies are needed to complement each other. With the progress of technical advancements, we can now explore and deduce how a relatively small number of FGFs act to assemble vast and intricate neuronal networks. Meanwhile, disorders of the FGF system are related to multiple neurological and psychiatric disorders [13]. Therefore, by figuring out the mechanism of the axonal pathway finding process steered by FGFs, we can have a greater depth of understanding of the pathological conditions related to the FGF system defects.

In this review, we first provide an overview of FGFs and discuss how FGFs are generated to exert their axon guidance functions after secretion. Then, a summary of the direct and indirect axon guidance effects of FGFs will be given. After that, the dose-dependent dual effects of FGFs on growth cones will be discussed. The next part of this review puts forward an understanding of the mechanisms and significance of FGF-FGFR interaction in axon guidance. Finally, we will describe the common cytoskeletal and downstream signaling mechanisms of axonal guidance, and summarize the major FGF’s intracellular signaling pathways in axon guidance.

## 2. Background of FGFs in the Development of Nervous System

FGFs, a broad family of polypeptide growth factors, were originally isolated and purified from the pituitary gland of bovines [14]. Many studies have discovered that FGFs have a variety of roles in the development of both vertebrates and invertebrates, and are extensively expressed in almost all cell types [15,16,17,18]. FGFs comprise approximately 200 amino acids, with a highly homologous core region of 120 amino acids [19]. The core region contains the binding site, which has an affinity for heparan sulfate proteoglycans (HPSGs) on cell surfaces or in the extracellular matrix [19]. Then, N and C sequences on the side of the core region enrich the diversity of FGF family members [20]. Acidic FGF (aFGF) and basic FGF (bFGF), commonly known as FGF1 and FGF2, are the first two FGFs isolated, purified and sequenced [21,22]. To date, 23 FGF members have been identified in different species. Through phylogenetic analysis, these FGFs are typically divided into seven subfamilies: FGF1, FGF4, FGF7, FGF8, FGF9, FGF11 and FGF15/19 [11]. While FGF15 in rodents is orthologous to FGF19 in other vertebrates, only 22 FGF family members can be found in these species when it comes to the FGF15/19 subfamily. Hence, not every species contains all FGF subtypes. Some studies have also proposed that there are eight FGF subfamilies, in which FGF3 is taken out of the FGF7 subfamily as a single subfamily with only one member. Generally, FGFs are divided into three groups according to their mechanisms of action: paracrine FGFs, endocrine FGFs and intracellular FGFs. Most FGFs are secreted outside the cells and bind to FGFRs to exert their effects. They usually act as autocrine, paracrine or juxtacrine factors, which are called canonical FGFs, except for three “hormone-like” FGF15/19 subfamily members (FGF15/19, FGF21 and FGF23) acting as endocrine factors [10]. Additionally, four intracellular members of the FGF11 subfamily (FGF11, FGF12, FGF13 and FGF14) are referred to as FGF homologous factors (FHFs) for not binding with FGFR, which are crucial controllers of cardiac and neural excitability.

Multiple studies have highlighted the importance of FGF expression in the nervous system as development progresses [10,23]. Although FGFs are widely secreted in almost every tissue and organ, the organizing centers of FGFs in the developing central nervous system are well-reported. For instance, FGF8, derived from the midbrain-hindbrain boundary (MHB), commonly known as the isthmic organizer (IsO), plays an instrumental role in assigning midbrain and hindbrain fates [24,25,26,27,28,29,30]. Moreover, FGF signaling in IsO functions does not appear to be limited to the orderly production of certain neuronal population types. In addition to mediating fate mapping activities, IsO FGF8 regulates the pathway finding of various axons at later stages in development, either in a direct manner, such as in trochlear motor axons, or in an indirect manner, such as in retinal ganglion cells (RGCs) axons, and also in repelling dopaminergic axons [31,32,33,34].

**Figure 1:**
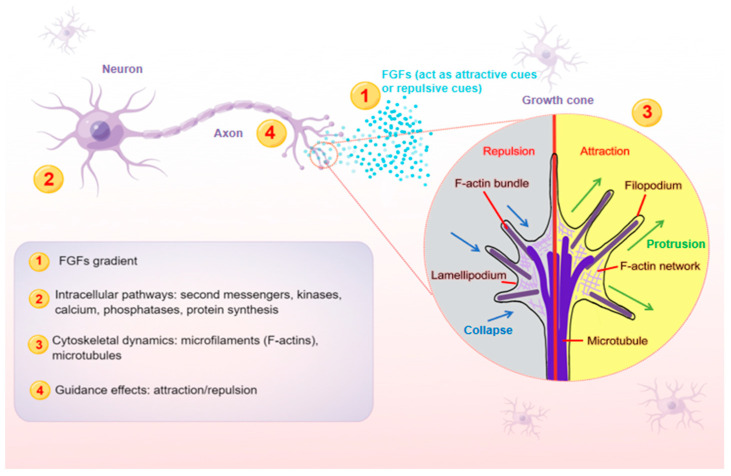
A schematic model illustrating the main steps (1–4) at which FGFs guide axons. Axon is a long and slender projection of a neuron, which is also defined as a nerve fiber. Axon guidance is an essential process for neural circuit formation. Growth cones at the tips of axons are crucial for detecting the guidance cues such as FGFs. Multiple guidance instructions then activate cellular responses, which recruit and assemble intracellular signaling components. By integrating diverse intracellular pathways, growth cones make corresponding cytoskeletal changes. Axons can thus exert appropriate motile actions.

In the nervous system, axons play an essential role in conducting electrical impulses (action potentials) from the body of neurons to their targets, such as muscles, neurons and glands. Therefore, it is crucial for neurons to stretch their axons along the correct paths to reach specific targets among myriad cells. To provide directional information for growing axons, the expressions of FGFs need to be restricted within a certain range of action, and then form a concentration gradient (Figure 1), exerting either attractive or repulsive effects in the proper axonal pathway [35]. As FGFs are secreted, they can bind to cofactors, such as HSPG cofactors or Klotho coreceptors, which are especially important for endocrine FGFs, to spatially limit their effects. HSPG cofactors control the diffusion and availability of secreted FGFs, regulating the effectiveness of axon guidance. FGFs are able to diffuse for some distance before becoming bound, which is thought to produce shallow gradients or provide long-range guidance signals. For example, due to their poor affinity for HSPGs, endocrine FGFs can diffuse into the circulation from the place of synthesis. In contrast, FGFs that are tethered right away after release tend to produce steep gradients that provide short-range guidance information. The interaction of FGFs with HSPGs in the extracellular matrix results in creating FGFs reservoirs and forming FGFs gradients, both of which are required for paracrine signaling [36].

## 3. Effects of FGFs on the Axon Guidance

FGFs within the developing neural system are necessarily required for patterning, neurogenesis and maintaining the physiology and homeostasis of neurons [37,38]. In addition to these fundamental activities, many studies have shown that FGFs, at the level of cells, act as guidance cues in the later development stages. It is obvious that FGFs can guide different axons to grow along their proper trajectory in various ways (Table 1).

### 3.1. Direct Axon Guidance Effects of FGFs in Vertebrates

Irving et al. found that FGF8 can make space for cerebellar development by inhibiting expressions of Hox genes in rhombomere 1, the anterior segment of the vertebrate hindbrain, which will develop into the cerebellum [48]. Inside this rhombomere within and posterior to the IsO, the trochlear motor neurons that innervated the eye muscle developed only in this restricted FGF8-positive area [48]. Studies on the formation of rhombomeres provide additional support for the involvement of FGF signaling in axon guidance by demonstrating how FGFs in the cerebellum function as synaptogenesis regulators and trochlear motor axon attractants [31,49]. In 2002, Irving and his partners showed that isthmic-derived FGF8 was also employed to direct trochlear axons out of the neural tube in chick embryos. They initially demonstrated how trochlear axons were drawn to FGF8-soaked beads in vitro, and how these beads implanted in vivo redirected the growth of those axons [31]. Before leaving the neural tube, FGF8 and isthmic tissue attracted the trochlear motor axons in the rostral hindbrain to turn dorsally and away from the floor plate of the isthmus, even though they could not significantly promote the growth of these axons. These axons’ trajectory is probably constrained along the segmental boundary of the MHB by FGF8 secreted along their route. Compared with many other classical chemoattractants, such as netrins, hepatocyte growth factor (HGF), and neurotrophins, FGFs exert a slightly distinct effect on trochlear axons because they cannot promote neurite outgrowth [50,51,52]. Furthermore, trochlear axons deviated from their correct pathway in explant cultures of the entire midbrain–hindbrain boundary area treated with the FGF8 inhibitor, FGFR blockers, or other defasciculation factors [53]. All these findings suggest that isthmic-derived FGF8 attracts trochlear axons, and plays a positive role in the formation of the IV cranial nerves.

In addition to the studies on trochlear motor axons, extensive research has shown the guidance roles of FGFs on other axons. For example, during the development of the cerebral cortex, FGF2 can elicit cortical pyramidal neurons’ interstitial axonal branching, leading to the collateral branching of layer five projection neurons extending to the destination [54]. Moreover, the dermomyotome expressed FGF8 when medial-class spinal motor neuron (MMCm) axons navigated toward this targeted interembryonic development [25,55]. Indeed, studies have demonstrated that FGF2, FGF4, and FGF9 were secreted in the dermomyotome at the moment of MMCm axonal pathway finding, suggesting that FGFs serve as important components guiding spinal motor neurons [56,57,58]. Finally, according to the results of Shirasaki’s research, FGF2, FGF4, FGF8, and FGF9 act as the axonal chemoattractants and the neurotrophins on MMCm axons in vitro [39,40]. Additionally, at early developmental stages, anosmin-1 encoded by the Kallmann syndrome gene (KAL-1) was essential for the differentiation and maturation of olfactory ensheathing cells (OEC) via the FGF2 signaling pathway, which could be blocked by the FGFR inhibitor SU5402 [59]. Then, at later embryonic and post-natal development, olfactory sensory axons crossed the nervous system boundary and targeted the olfactory bulb (OB). This pathway-finding process depends on the proper glial environment produced by OEC wrapping the olfactory sensory axons. Studies have demonstrated that FGF1 expressed by OEC regulates the olfactory sensory axon growth between the olfactory neuroepithelium and the OB, which suggests that FGF1 is essential in forming the olfactory pathway [60].

### 3.2. Indirect Axon Guidance Effects of FGFs in Vertebrates

Recently, many data points suggest that FGFs can also guide the pathway finding of axons indirectly by patterning other guidance cues. Retinal ganglion cell (RGC) axons migrate from the eye to their primary contralateral destination in the brain, the optic tectum, during the development of the visual system. Additionally, in the posterior optic tectum at the IsO, several secreted FGFs act as guidance cues. Many previous pieces of research have proved that FGF2 regulates the growth and guidance of RGC axons at later stages of development in vivo [61,62,63]. Webber’s laboratory has previously confirmed that RGC axons were directly repelled by FGF2 both in vivo and in vitro [46]. Consistently, Song et al. have also found that FGFs acted as repellents to guide RGC axons away from the mid-diencephalon and towards the optic tectum [64]. Whereas, while RGC axons were repelled by FGF8 ectopically injected into the axonal trajectory in vivo, this repulsive response was not observed in vitro. It was possible that FGF8 indirectly directed RGC axons in vivo by inducing neuroepithelial cells to secrete a component that repelled these axons [59]. It has been demonstrated that FGF8 controlled the synthesis of the transcription factor engrailed-2, which was generated in the mesencephalon along a decreasing gradient from caudal to rostral [65]. Under the positive regulation of engrailed-2, the two Eph receptor tyrosine kinase ligands (ELF-1 and RAGS) were likewise expressed in a decreasing caudal-to-rostral gradient throughout the optic tectum and then controlled the patterning of retinal axon terminals [66]. Another compelling case is that FGF8 can indirectly repel the axons of midbrain dopaminergic neurons (mDAN) extending through the diencephalon. How FGF8 regulates the rostro-caudal growth polarity of mDAN axons has been proven by inducing the expression of the chemorepellent semaphorin 3F (sema3F) in the midbrain [45]. Moreover, in the previous in vitro study, FGF10 enhanced the outgrowth of SAG neurites and maintained these neurons’ survival, which could not be inhibited by SU5402. Therefore, additional signaling pathways in this development process may be indirectly activated by FGF10 [41]. Furthermore, FGF3 and FGF8-dependent FGF22 signaling has been found to indirectly change the cytoskeleton during axon guidance and shape the development of the midbrain, which may act by regulating WNT1 [67,68]. Additionally, the indirect guidance method may explain the results that, despite lacking FGFR1 signaling, some transplanted ES cell-derived MMCm motoneurons appropriately projected to epaxial muscles [69]. Overall, these indirect guidance fashions significantly contribute to the variety and complexity of FGFs’ activities in axonal pathfinding and the subsequent development of the nervous system.

### 3.3. FGF’s Axon Guidance Functions in Invertebrates

FGF signaling is also proven to participate in the invertebrate nervous system, including the axonal guidance process. FGF2 expressed in the head of the cockroach embryos in vivo served as a crucial antagonist of an axon growth inhibitor generated in the thorax, which was essential in guiding the pioneer axonal pathway to turn and elongate proximally in the coxa or into the CNS [18]. In addition, FGF signaling indirectly guided the proper extension of axons and maintained the correct position of different classifications of axons in *Caenorhabditis elegans* [70]. Furthermore, the heartless, which is the fly homolog of the vertebrate FGFR, is required for the outgrowth of axons in cultured Drosophila neurons [71]. Nonetheless, the roles of FGFs in the axonal pathway-finding process in invertebrates have not been extensively explored. 

## 4. Concentration-Dependent Responses of Growth Cones to FGFs

### 4.1. Continuous Changed Responses of the Same Axons over Time and Space

At different developmental stages, the surrounding and internal environments of the same axons undergo continuous changes, which lead to more complex guidance effects of FGFs. Many researchers found that FGFs may have a dose-dependent dual function during the axon guidance process (Table 1). The patterning function of FGFs begins at very early stages of development, so it is reasonable to speculate that the remaining established gradients of FGFs play a significant role in establishing the complicated neuronal connectivity of the nervous system. Previous research has found that the misrouting of thalamocortical axons (TCAs) could result from early ectopic sources of FGF8 [72]. The guidance of TCAs is also directed by other FGFs, which may exert bifunctional guidance roles. For example, the low concentration of FGF10 and FGF3 attracts TCAs; in contrast, high levels of FGF3 and FGF10 in the hypothalamus can serve as a chemorepellent, directing TCAs into the ventral telencephalon and away from the hypothalamus [43,73]. Furthermore, a previous study demonstrated that FGF3 and FGF10, the FGF7 subfamily members, can not only regulate the differentiation of midline-derived progenitor cells during the hypothalamic infundibular development, but also guide the hypothalamic axons to the median eminence in the later stage of hypothalamic development bifunctionally [44,74]. The research showed that low-concentration FGF3 and FGF10 attracted hypothalamo-neurohypophyseal (H-NH) axons, while high-concentration FGF3 and FGF10 repelled H-NH axons [44]. These studies demonstrate that FGF3 and FGF10 exert concentration-dependent effects on the diencephalic axon guidance, helping axons navigate to their appropriate destinations. In the study by Kristen et al., they found that only low-concentration FGF8, FGF10, and FGF19 could significantly promote chicken statoacoustic ganglion (SAG) axon asymmetric outgrowth in vitro [41]. Additionally, the axonal guidance effects of FGFs are not only concentration-dependent, but also time-specific. Previous studies have proved that FGF2 enhances the survival of chick SAG neurons in vitro, but only in the early (E2–3) and later (E8–16) stages [75,76]. In contrast, in Kristen’s study, only E4 SAG axons did not respond to the bioactivity of FGF2 [41]. Thus, the responses of chick SAG to FGF2 may be time-sensitive [76,77].

### 4.2. The Molecular Mechanism behind the Concentration-Dependent Responses

It is unnecessary to categorize any guidance cue as either attractive or repulsive since it behaves differently in different circumstances. Firstly, the response of an axon to a given guidance cue will be influenced by other crosstalk cues in the surrounding environment. At the tips of growing axons, growth cones are identified as the highly motile sensory apparatus that can respond to extrinsic guidance instructions and then guide axons to their specific targets [53]. According to the embryological research concentrating on the topographic projections of spinal motor axons, distinct axon pathfinding of motor neuron subtypes differed in their intrinsic capabilities. The finding shows that motor axons have been genetically preprogrammed to detect guidance cues before they reach their targets [78]. Hence, based on the diverse complement of expressed receptors, the same guidance cue can be interpreted variously by distinct neurons. Secondly, the responses of axons to the guidance cues need to be plastic over space and time, and much research has shown that intracellular signaling systems in neurons also critically affect the roles of guidance cues (Figure 1). Cyclic nucleotide levels (cAMP and cGMP activity) and intracellular calcium concentration are demonstrated to be two key regulators participating in modulating the guidance responses [5,79,80,81,82]. Elevation of intracellular calcium can convert repulsion into attraction [82]. Moreover, the effect of the cAMP-dependent or cGMP-dependent pathways can be mediated by protein kinase A (PKA) or protein kinase G (PKG), which can modulate the synthesis of cytoskeleton-associated proteins [83]. Many experiments have confirmed that reducing cAMP or cGMP levels switches attraction to repulsion, while increasing cAMP or cGMP favors converting a repulsive response to an attractive one [42,80,82,84]. However, the level of cGMP in dendrites may be higher than in axons because the guanylyl cyclase is only detected in dendrites [85]. Consistent with the mechanism of many guidance cues, studies suggested that cAMP and other regulating molecules were crucially important in the concentration-dependent responses of growth cones to FGFs over space and time [42,80,85].

## 5. FGFs and FGFRs Interaction

### 5.1. Why FGFs Exert Distinct Effects on Different Axons

There are two basic theories explaining the different effects of FGFs on axons. In the guiding process, the FGF-FGFR system is of great concern. The first theory indicates that different FGF-FGFR combinations activate different intracellular signaling pathways, considering that different FGF ligands and receptors can send distinct signals to cells. Another theory highlights that the modulation and interpretation of FGF signaling are determined by the target cell types and their surroundings. For instance, one type of cell responds to FGFs by activating a particular intracellular pathway, whereas another type of cell responds by using a divergent intracellular mechanism. Although it is doubtless that both of these main mechanisms will be discovered to impact FGF functions in vivo, most of the available data support the hypothesis that the mechanism described in the second theory plays a dominant role in most cases. Different types of FGFRs share about 46% amino acid identity. The high degree of homology leads to quite similar signaling pathways between different FGFRs [86]. Additionally, using chimeric receptors made of the cytoplasmic domains of FGFR1, FGFR3, or FGFR4 linked to the extracellular domain of the PDGF receptor, studies have demonstrated that the main distinction between various FGFRs was the strength of tyrosine kinase activity but not the variation of target proteins [87]. Thus, all FGFR subtypes activate downstream signaling cascades at varying intensities. This mechanism leads to changes in cell responses that are either quantitative or qualitative [36,88].

### 5.2. The Importance of the FGF-FGFR System in Axon Pathfinding

FGFRs are a group of highly conserved membrane-bound receptors. They harbor four main members in vertebrates (FGFR1, FGFR2, FGFR3, and FGFR4), two in Drosophila (heartless and breathless), and one in Caenorhabditis elegans (egl-15). Although the FGF family has been found to contain at least 23 members up to now, only 18 secreted FGFs act as FGFR ligands, and then initiate a series of intracellular signaling cascades. The intracellular FGFs are not secreted and act independently of FGFRs. They always engage with the intracellular regions of voltage-gated sodium channels or remain in the nucleus [89]. FGFRs are single-spanning transmembrane proteins. Significant variation exists in the affinities of FGFRs for their FGF ligands. Three immunoglobulin domains (I, II, and III) found in the extracellular domain of FGFRs interact with HPSGs and FGF ligands [90]. The isoforms IIIa, IIIb, and IIIc are produced specifically by alternative splicing in the second part of the third immunoglobulin loop, and they have the strongest influence on the specificity of FGF receptor binding [86]. The split intracellular domain has the receptor tyrosine kinases (RTKs) activity and is involved in the interaction with signaling molecules and intracellular substrates [91]. At first, heparinases, proteases, or certain FGF-binding proteins can release FGFs from the extracellular matrix. Then, the released FGFs bind to cell surface HPSGs and create a ternary complex, which can prevent FGFs from being degraded by proteases and restrict their diffusion, stabilizing the FGF-FGFR association [92]. It is noteworthy that the FGFs and FGFRs can also interact with extracellular molecules, such as cell adhesion molecules (CAMs), cadherins, integrins, and fibronectin [93]. Therefore, more complex and refined considerations are needed when exploring the mechanism of the action of FGF-FGFR interaction.

FGFRs are distributed widely in the nervous system (Table 2). Undoubtedly, FGF-FGFR signaling disturbances could lead to deficiencies in axon guidance, which could eventually result in developmental and behavioral aberrations. However, overlapping expression patterns of FGFs conceal individual roles of FGFs, because they always functionally compensate for one another at various phases of development and in various tissues. But with relatively small numbers of FGFRs, it will be easier to design knock-out or knock-down experiments, and prevent compensation when certain FGF signaling is disrupted. The nervous system contains the highest concentration of FGFR1, which is primarily expressed by neurons, astrocytes, and radial glia [94]. In adult mice, the disruption of FGFR1 caused the death of certain midline glial cells, which prevented callosal and other axons from crossing the midline and connecting the neocortex of the two hemispheres [95]. Previous studies have shown that the growth cone and cell body of RGCs express FGFR1 from the very beginning of development [96]. Furthermore, further research showed that FGFR1 signaling was crucial for the elongation and early target recognition of growing RGC axons, which helped to form the vertebrate visual system [61,63]. In addition, Shirasaki discovered that MMCm cells expressed FGFR1 specifically when their axons extended toward the dermomyotome [40]. Consistent with Ornitz’s 1996 research findings, the FGFR1 ligands FGF2, FGF4, FGF8, and FGF9 strongly promoted the outgrowth of motor axons, while the FGFR2 ligand FGF10 did not exert relevant functions [40,97]. However, recent studies suggest that the additional FGFRs, such as FGFR3, may be involved in the navigation of ES cell-derived MMCm axons [69,98]. Additionally, it has been proposed by knock-down studies that in zebrafish, FGFR1 serves as the main receptor for FGF signals coming from the IsO [59]. FGFR1 and FGFR2 were required for early embryonic development, according to studies of mice with null mutations in each of the FGFR genes. These studies also demonstrate that these two FGFRs are crucial for the neurogenesis and precursor proliferation. Nonetheless, the FGFR3-deficient mice are capable of survival, and appear to have no evident telencephalic abnormalities [12]. Additionally, a previous study has shown that FGFR3 is necessary for correct cortex formation [99].

## 6. Common Downstream Signaling Mechanisms

### 6.1. Three Major FGF Signaling Pathways

**Figure 2:**
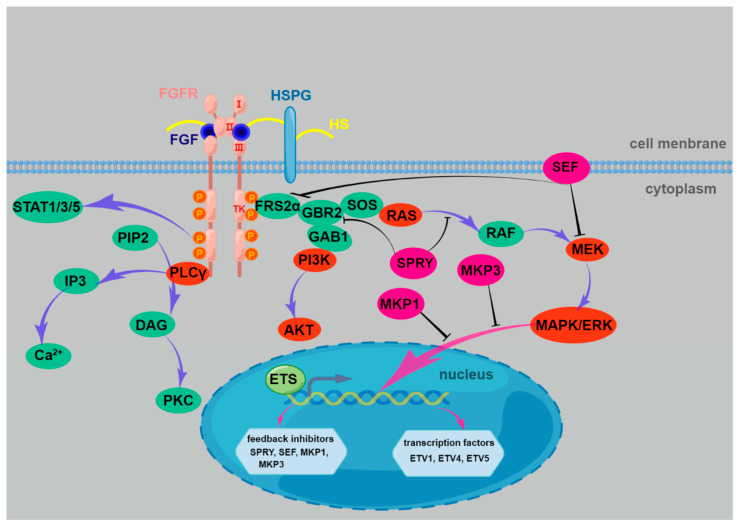
Three major FGF intracellular signaling pathways. Upon ligand binding, receptor dimmers are formed and their intrinsic tyrosine kinase is activated, resulting in phosphorylation of multiple tyrosine residues on the receptors. These phosphorylated tyrosine residues then serve as docking sites for docking proteins or signaling enzymes. Thus, signaling complexes are assembled and recruited to the active receptors, resulting in a cascade of phosphorylation events. Multiple signal transduction pathways can be activated by FGFs, but among them, three pathways are well studied. The best understood are FRS2-Ras-MAP kinase pathways, which can be observed in almost all cell types; the cell type-dependent PI3 kinase-AKT pathway and the PLCγ pathways.

The combination of FGFs and FGFRs dimerizes the receptors and activates the tyrosine kinases. Then, the intracellular domain of the receptor becomes autophosphorylated and the signaling components are recruited and assembled. The activated FGFR mainly phosphorylates adaptor proteins for three major intracellular signaling pathways, RAS-MAPK-ERK, PI3K-AKT and PLCγ [15,104,105,106] (Figure 2). Among these three major signaling pathways, the most regular one used by FGFRs is the MAPK signaling cascade, which plays a critical role in tissue patterning, including stem cell proliferation, differentiation, and axon guidance. For instance, activation of FGFR1 selectively activates downstream MAPK/ERK, directing the attraction of MMCm axons mediated by FGF8 [76]. The PLCγ pathway, a different cascade, is also proven to be highly significant for the axonal pathway-finding process. It has been proved that PLCγ can mediate a RGC repellent effect induced by FGF2 [107]. Meanwhile, acting as a downstream pathway for FGFs or for other neurotrophic factors, the PI3K pathway regulates various aspects of neural development, including axon development [108,109,110,111]. For example, by triggering the PI3K pathway, nerve growth factor (NGF) facilitated branch development in sensory neurons [112]. Additionally, by engaging both the MAPK and PI3K pathways, brain-derived neurotrophic factor (BDNF) promotes the outgrowth of sympathetic axons [113]. The coactivation of the PLCγ and PI3K pathways was necessary for the growth cones of Xenopus spinal neurons to turn in response to netrin-1 gradients [114]. Additionally, prominent differences are found between distinct FGFR signaling pathways, which may occur as a result of variations in ligand activation and intracellular receptor transport cascades. For instance, FGFR1 activates ERK and PLCγ more potently than FGFR4. Additionally, FGFR1 rather than FGFR2 is the cause of higher ERK activation [115].

### 6.2. Common Cytoskeletal Alterations

FGFs’ intracellular signaling pathway is necessary for regulating diverse developmental processes of the brain, including neural induction, patterning and axon navigation [23,58]. Several studies have investigated the intracellular signaling pathways downstream of FGFs-mediated axonal pathway findings. Cytoskeletal alterations in the axons are the intuitive reflection of the activation of the corresponding signaling pathways [116]. Axon extension and guidance are the basis of the pathway finding process, in which filopodia’s actin cytoskeleton generally determines axon guidance effects, attractive or repulsive, while axon extension is related to microtubules [81,117,118]. Due to their limited intracellular volume, filopodia are particularly susceptible to external cues, so they always extend asymmetrically before the entire growth cone turns [119]. Therefore, without filopodia, growth cones cannot properly respond to guidance cues, resulting in the disoriented growth of axons. Dynamic microtubules preferentially develop along filopodial actin filaments, and the stabilization or capture of microtubule bundles in the growing filopodium may be a crucial step in the turning of the growth cone [120,121]. Local microtubule stabilization on one side of the growth cone causes attractive steering. However, depolymerization of the local microtubule, on the other hand, promotes growth cones to diverge. All these illustrate microtubules’ crucial functions in growth cone directional guiding (Figure 1).

### 6.3. Recruitment and Convergence of FGF Signaling Pathways

The coexisting intracellular signaling pathways need to converge on some ultimate cytoplasmic effectors. Thus, directed neurite extension with the asymmetric cytoskeletal reconfiguration result in attractive or repulsive axonal. Webber et al. discovered that the MAPK, PLCγ and phosphatidylcholine phospholipase C (PC-PLC) pathways were all required for FGF2 to stimulate RGC neurite extension in a convergent fashion in vitro, and the repulsive effect of FGF2 on the RGC neurite guidance was only mediated by these two PLC pathways [47]. In contrast, the PI3K pathway was not necessary for this axon growth and guidance process [47]. Thus, the MAPK and two PLC pathways may work together to regulate microtubule polymerization downstream of FGF2 in RGC axons. Given that FGF2 repelled RGC growth cones via two PLC pathways, they are most likely to depolymerize the actin on the side of the growth cone nearest to the FGF2. Combined with the preceding discussion about MMCm axons guidance, the results of Soundararajan’s experiments indicated that the turning response of these axons to FGF8 was conducted by the FGFR1-downstream MAPK pathway [69]. It appeared that the PI3K pathway was also not involved in this process [69]. Yang J.J. et al. found that despite the differences in FGFR dependency, PI3K pathway is likely a shared downstream regulator to maintains slit1 and semaphorin 3a (sema3a) repellents expression, which took part in RGC axon guidance [111]. In accordance with the results of Webber’s study, this process depended on PI3K signaling in brain neuroepithelial cells but not PI3K signaling in the RGC growth cones [47,111]. Considering the variability and complexity of time and space during the axon guidance process, distinct downstream signaling pathways in response to a chemoattractant or a chemorepellent of FGFs should be further explored.

## 7. Summary and Perspective

The extraordinary axon guidance effects of FGFs lead to astonishingly complex patterns of neuronal wiring, which are determined by an FGF-FGFR signaling system. The guidance roles of FGFs can be modulated by many factors, including different combinations of FGF ligands and receptors and complex external and/or internal conditions of growth cones. Similar to other diffusible guidance molecules, FGFs are also bifunctional. The growth cone’s reaction to a specific FGF signal can change over space and time, which is concentration-dependent and can be switched by cAMP and its downstream molecules. The MAPK, PLCγ, PC-PLC and PI3K downstreams are major FGF signaling pathways during the axon guidance process. However, to form the delicate neuronal wiring, numerous molecules must be integrated with growth cones, and no one signal should be taken into account separately. Thus, the intracellular signaling triggered by FGFs and other guidance cues needs to converge into a set of common cytoplasmic effectors. However, it is still unclear why distinct neuronal populations employ various signaling routes to control development. We still have a limited and superficial grasp of how FGFs perform so many axon-guiding roles. It is unclear how FGFs interact with other signaling pathways and converge into an ultimate instruction to determine axonal cytoskeleton behaviors, so the need is now greater than ever to push ahead with analysis of molecular mechanisms of FGF guidance effects in vitro and in vivo. Together, all these studies move forward to draw a more established picture of how a comparatively small number of FGFs molecules generate such astonishingly complex patterns of neuronal wiring. Additionally, the FGF signaling deregulation is regarded as a driver in some neural diseases. Therefore, clinical applications of FGFs in neurological regeneration and psychiatric disorders are thought to be interesting and challenging research orientations in the future.

## Figures and Tables

**Table 1:** Guidance effects of FGFs on axons in vertebrates.

Axons	FGFs	Effects	Note	References
Medial motor column (MMCm) axons	FGF2, 4, 8, 9	Attractive	Directly	[39,40]
Statoacoustic ganglion (SAG) neurites	FGF8, 10, 19	Promote asymmetric outgrowth	Directly	[41]
Thalamocortical axons (TCA)	FGF3, 10	Low concentration: attractive	Directly	[42,43]
		High concentration: repulsive		
Hypothalamic axons	FGF3, 10	Low concentration: attractive	Directly	[44]
		High concentration: repulsive		
Midbrain dopaminergic neuron (mDAN) axons	FGF8	Repulsive	Indirectly	[45]
Trochlear motor axons	FGF8	Attractive	Directly	[31]
Retinal ganglion cell (RGC) axons	FGF2	Repulsive	Directly	[46,47]
RGC axons	FGF8	Repulsive	Indirectly	[33]

**Table 2:** FGFRs’ distributions in the nervous system.

FGFRs	Main Sites	Expression Phases
FGFR1	Neurons [100,101]	In embryonic and adult development [97,102]
FGFR2	Oligodendrocytes [100,101]	In embryonic and adult development [97,102]
FGFR3	Astrocytes [101]	In embryonic and adult development [97,102]
FGFR4	Neurons [101]	Only in embryonic development but not in the adult brain apart from the lateral habenular nucleus [103]

## Data Availability

The data presented in this study are available in article.

## References

[1] Seiradake E., Jones E.Y., Klein R. (2016). Structural Perspectives on Axon Guidance. Annu. Rev. Cell Dev. Biol..

[2] Marin O., Valiente M., Ge X., Tsai L.H. (2010). Guiding neuronal cell migrations. Cold Spring Harb. Perspect. Biol..

[3] O’Donnell M., Chance R.K., Bashaw G.J. (2009). Axon growth and guidance: Receptor regulation and signal transduction. Annu. Rev. Neurosci..

[4] Tessier-Lavigne M., Goodman C.S. (1996). The molecular biology of axon guidance. Science.

[5] McFarlane S., Holt C.E. (1997). Growth factors: A role in guiding axons?. Trends Cell Biol..

[6] Yam P.T., Charron F. (2013). Signaling mechanisms of non-conventional axon guidance cues: The Shh, BMP and Wnt morphogens. Curr. Opin. Neurobiol..

[7] Kiecker C., Lumsden A. (2004). Hedgehog signaling from the ZLI regulates diencephalic regional identity. Nat. Neurosci..

[8] Bluske K.K., Vue T.Y., Kawakami Y., Taketo M.M., Yoshikawa K., Johnson J.E., Nakagawa Y. (2012). β-Catenin signaling specifies progenitor cell identity in parallel with Shh signaling in the developing mammalian thalamus. Development.

[9] Vue T.Y., Bluske K., Alishahi A., Yang L.L., Koyano-Nakagawa N., Novitch B., Nakagawa Y. (2009). Sonic hedgehog signaling controls thalamic progenitor identity and nuclei specification in mice. J. Neurosci..

[10] Ornitz D.M., Itoh N. (2015). The Fibroblast Growth Factor signaling pathway. Wiley Interdiscip. Rev. Dev. Biol..

[11] Itoh N. (2007). The Fgf families in humans, mice, and zebrafish: Their evolutional processes and roles in development, metabolism, and disease. Biol. Pharm. Bull..

[12] Klimaschewski L., Claus P. (2021). Fibroblast Growth Factor Signalling in the Diseased Nervous System. Mol. Neurobiol..

[13] Turner C.A., Eren-Kocak E., Inui E.G., Watson S.J., Akil H. (2016). Dysregulated fibroblast growth factor (FGF) signaling in neurological and psychiatric disorders. Semin. Cell Dev. Biol..

[14] Gospodarowicz D. (1975). Purification of a fibroblast growth factor from bovine pituitary. J. Biol. Chem..

[15] Goetz R., Mohammadi M. (2013). Exploring mechanisms of FGF signalling through the lens of structural biology. Nat. Rev. Mol. Cell Biol..

[16] Ornitz D.M., Itoh N. (2001). Fibroblast growth factors. Genome Biol..

[17] Yun Y.R., Won J.E., Jeon E., Lee S., Kang W., Jo H., Jang J.H., Shin U.S., Kim H.W. (2010). Fibroblast growth factors: Biology, function, and application for tissue regeneration. J. Tissue Eng..

[18] Nyhus J.K., Denburg J.L. (1998). The in vivo regulation of pioneer axon growth by FGF-2 and heparan sulfate proteoglycans in cultured embryos of the cockroach. Mol. Cell. Neurosci..

[19] Powers C.J., McLeskey S.W., Wellstein A. (2000). Fibroblast growth factors, their receptors and signaling. Endocr. Relat. Cancer.

[20] Mohammadi M., Olsen S.K., Ibrahimi O.A. (2005). Structural basis for fibroblast growth factor receptor activation. Cytokine Growth Factor Rev..

[21] Abraham J.A., Mergia A., Whang J.L., Tumolo A., Friedman J., Hjerrild K.A., Gospodarowicz D., Fiddes J.C. (1986). Nucleotide sequence of a bovine clone encoding the angiogenic protein, basic fibroblast growth factor. Science.

[22] Abraham J.A., Whang J.L., Tumolo A., Mergia A., Friedman J., Gospodarowicz D., Fiddes J.C. (1986). Human basic fibroblast growth factor: Nucleotide sequence and genomic organization. EMBO J..

[23] Baird A. (1994). Fibroblast growth factors: Activities and significance of non-neurotrophin neurotrophic growth factors. Curr. Opin. Neurobiol..

[24] Heikinheimo M., Lawshe A., Shackleford G.M., Wilson D.B., MacArthur C.A. (1994). Fgf-8 expression in the post-gastrulation mouse suggests roles in the development of the face, limbs and central nervous system. Mech. Dev..

[25] Crossley P.H., Martin G.R. (1995). The mouse Fgf8 gene encodes a family of polypeptides and is expressed in regions that direct outgrowth and patterning in the developing embryo. Development.

[26] Crossley P.H., Martinez S., Martin G.R. (1996). Midbrain development induced by FGF8 in the chick embryo. Nature.

[27] Chi C.L., Martinez S., Wurst W., Martin G.R. (2003). The isthmic organizer signal FGF8 is required for cell survival in the prospective midbrain and cerebellum. Development.

[28] Basson M.A., Echevarria D., Ahn C.P., Sudarov A., Joyner A.L., Mason I.J., Martinez S., Martin G.R. (2008). Specific regions within the embryonic midbrain and cerebellum require different levels of FGF signaling during development. Development.

[29] Chen Y., Mohammadi M., Flanagan J.G. (2009). Graded levels of FGF protein span the midbrain and can instruct graded induction and repression of neural mapping labels. Neuron.

[30] Xu J., Liu Z., Ornitz D.M. (2000). Temporal and spatial gradients of Fgf8 and Fgf17 regulate proliferation and differentiation of midline cerebellar structures. Development.

[31] Irving C., Malhas A., Guthrie S., Mason I. (2002). Establishing the trochlear motor axon trajectory: Role of the isthmic organiser and Fgf8. Development.

[32] Nakamura S., Ito Y., Shirasaki R., Murakami F. (2000). Local directional cues control growth polarity of dopaminergic axons along the rostrocaudal axis. J. Neurosci..

[33] Nakamura H., Sugiyama S. (2004). Polarity and laminar formation of the optic tectum in relation to retinal projection. J. Neurobiol..

[34] Volkmann K., Chen Y.Y., Harris M.P., Wullimann M.F., Koster R.W. (2010). The zebrafish cerebellar upper rhombic lip generates tegmental hindbrain nuclei by long-distance migration in an evolutionary conserved manner. J. Comp. Neurol..

[35] Zou Y., Lyuksyutova A.I. (2007). Morphogens as conserved axon guidance cues. Curr. Opin. Neurobiol..

[36] Ornitz D.M. (2000). FGFs, heparan sulfate and FGFRs: Complex interactions essential for development. Bioessays.

[37] Dorey K., Amaya E. (2010). FGF signalling: Diverse roles during early vertebrate embryogenesis. Development.

[38] Itoh N., Ornitz D.M. (2011). Fibroblast growth factors: From molecular evolution to roles in development, metabolism and disease. J. Biochem..

[39] Stifani N. (2014). Motor neurons and the generation of spinal motor neuron diversity. Front. Cell. Neurosci..

[40] Shirasaki R., Lewcock J.W., Lettieri K., Pfaff S.L. (2006). FGF as a target-derived chemoattractant for developing motor axons genetically programmed by the LIM code. Neuron.

[41] Fantetti K.N., Fekete D.M. (2012). Members of the BMP, Shh, and FGF morphogen families promote chicken statoacoustic ganglion neurite outgrowth and neuron survival in vitro. Dev. Neurobiol..

[42] Liu K., Lv Z., Huang H., Yu S., Xiao L., Li X., Li G., Liu F. (2020). FGF3 from the Hypothalamus Regulates the Guidance of Thalamocortical Axons. Dev. Neurosci..

[43] Liu K., Lv Z., Huang H., Li M., Xiao L., Li X., Li G., Liu F. (2020). FGF10 regulates thalamocortical axon guidance in the developing thalamus. Neurosci. Lett..

[44] Liu F., Pogoda H.M., Pearson C.A., Ohyama K., Lohr H., Hammerschmidt M., Placzek M. (2013). Direct and indirect roles of Fgf3 and Fgf10 in innervation and vascularisation of the vertebrate hypothalamic neurohypophysis. Development.

[45] Yamauchi K., Mizushima S., Tamada A., Yamamoto N., Takashima S., Murakami F. (2009). FGF8 signaling regulates growth of midbrain dopaminergic axons by inducing semaphorin 3F. J. Neurosci..

[46] Webber C.A., Hyakutake M.T., McFarlane S. (2003). Fibroblast growth factors redirect retinal axons In Vitro and In Vivo. Dev. Biol..

[47] Webber C.A., Chen Y.Y., Hehr C.L., Johnston J., McFarlane S. (2005). Multiple signaling pathways regulate FGF-2-induced retinal ganglion cell neurite extension and growth cone guidance. Mol. Cell. Neurosci..

[48] Irving C., Mason I. (2000). Signalling by FGF8 from the isthmus patterns anterior hindbrain and establishes the anterior limit of Hox gene expression. Development.

[49] Partanen J. (2007). FGF signalling pathways in development of the midbrain and anterior hindbrain. J. Neurochem..

[50] Ebens A., Brose K., Leonardo E.D., Hanson M.G., Bladt F., Birchmeier C., Barres B.A., Tessier-Lavigne M. (1996). Hepatocyte growth factor/scatter factor is an axonal chemoattractant and a neurotrophic factor for spinal motor neurons. Neuron.

[51] O’Connor R., Tessier-Lavigne M. (1999). Identification of maxillary factor, a maxillary process-derived chemoattractant for developing trigeminal sensory axons. Neuron.

[52] Serafini T., Kennedy T.E., Galko M.J., Mirzayan C., Jessell T.M., Tessier-Lavigne M. (1994). The netrins define a family of axon outgrowth-promoting proteins homologous to C. elegans UNC-6. Cell.

[53] Bovolenta P. (2005). Morphogen signaling at the vertebrate growth cone: A few cases or a general strategy?. J. Neurobiol..

[54] Szebenyi G., Dent E.W., Callaway J.L., Seys C., Lueth H., Kalil K. (2001). Fibroblast growth factor-2 promotes axon branching of cortical neurons by influencing morphology and behavior of the primary growth cone. J. Neurosci..

[55] Mahmood R., Bresnick J., Hornbruch A., Mahony C., Morton N., Colquhoun K., Martin P., Lumsden A., Dickson C., Mason I. (1995). A role for FGF-8 in the initiation and maintenance of vertebrate limb bud outgrowth. Curr. Biol..

[56] Niswander L., Martin G.R. (1992). Fgf-4 expression during gastrulation, myogenesis, limb and tooth development in the mouse. Development.

[57] Colvin J.S., Bohne B.A., Harding G.W., McEwen D.G., Ornitz D.M. (1996). Skeletal overgrowth and deafness in mice lacking fibroblast growth factor receptor 3. Nat. Genet..

[58] Thisse B., Thisse C. (2005). Functions and regulations of fibroblast growth factor signaling during embryonic development. Dev. Biol..

[59] Atkinson-Leadbeater K., Bertolesi G.E., Hehr C.L., Webber C.A., Cechmanek P.B., McFarlane S. (2010). Dynamic expression of axon guidance cues required for optic tract development is controlled by fibroblast growth factor signaling. J. Neurosci..

[60] Key B., Treloar H.B., Wangerek L., Ford M.D., Nurcombe V. (1996). Expression and localization of FGF-1 in the developing rat olfactory system. J. Comp. Neurol..

[61] Brittis P.A., Silver J., Walsh F.S., Doherty P. (1996). Fibroblast growth factor receptor function is required for the orderly projection of ganglion cell axons in the developing mammalian retina. Mol. Cell. Neurosci..

[62] McFarlane S., McNeill L., Holt C.E. (1995). FGF signaling and target recognition in the developing Xenopus visual system. Neuron.

[63] McFarlane S., Cornel E., Amaya E., Holt C.E. (1996). Inhibition of FGF receptor activity in retinal ganglion cell axons causes errors in target recognition. Neuron.

[64] Song Y., Li D., Farrelly O., Miles L., Li F., Kim S.E., Lo T.Y., Wang F., Li T., Thompson-Peer K.L. (2019). The Mechanosensitive Ion Channel Piezo Inhibits Axon Regeneration. Neuron.

[65] Lee S.M., Danielian P.S., Fritzsch B., McMahon A.P. (1997). Evidence that FGF8 signalling from the midbrain-hindbrain junction regulates growth and polarity in the developing midbrain. Development.

[66] Logan C., Wizenmann A., Drescher U., Monschau B., Bonhoeffer F., Lumsden A. (1996). Rostral optic tectum acquires caudal characteristics following ectopic engrailed expression. Curr. Biol..

[67] Ciani L., Salinas P.C. (2005). WNTs in the vertebrate nervous system: From patterning to neuronal connectivity. Nat. Rev. Neurosci..

[68] Miyake A., Itoh N. (2013). Fgf22 regulated by Fgf3/Fgf8 signaling is required for zebrafish midbrain development. Biol. Open.

[69] Soundararajan P., Fawcett J.P., Rafuse V.F. (2010). Guidance of postural motoneurons requires MAPK/ERK signaling downstream of fibroblast growth factor receptor 1. J. Neurosci..

[70] Bulow H.E., Boulin T., Hobert O. (2004). Differential functions of the C. elegans FGF receptor in axon outgrowth and maintenance of axon position. Neuron.

[71] Forni J.J., Romani S., Doherty P., Tear G. (2004). Neuroglian and FasciclinII can promote neurite outgrowth via the FGF receptor Heartless. Mol. Cell. Neurosci..

[72] Shimogori T., Grove E.A. (2005). Fibroblast growth factor 8 regulates neocortical guidance of area-specific thalamic innervation. J. Neurosci..

[73] Braisted J.E., Ringstedt T., O’Leary D.D. (2009). Slits are chemorepellents endogenous to hypothalamus and steer thalamocortical axons into ventral telencephalon. Cereb. Cortex.

[74] Pearson C.A., Ohyama K., Manning L., Aghamohammadzadeh S., Sang H., Placzek M. (2011). FGF-dependent midline-derived progenitor cells in hypothalamic infundibular development. Development.

[75] Hossain W.A., Zhou X., Rutledge A., Baier C., Morest D.K. (1996). Basic fibroblast growth factor affects neuronal migration and differentiation in normotypic cell cultures from the cochleovestibular ganglion of the chick embryo. Exp. Neurol..

[76] Carnicero E., Garrido J.J., Alonso M.T., Schimmang T. (2001). Roles of fibroblast growth factor 2 during innervation of the avian inner ear. J. Neurochem..

[77] Hossain W.A., Brumwell C.L., Morest D.K. (2002). Sequential interactions of fibroblast growth factor-2, brain-derived neurotrophic factor, neurotrophin-3, and their receptors define critical periods in the development of cochlear ganglion cells. Exp. Neurol..

[78] Landmesser L.T. (1992). Growth Cone Guidance in the Avian Limb: A Search for Cellular and Molecular Mechanisms.

[79] Gomez T.M., Spitzer N.C. (1999). In vivo regulation of axon extension and pathfinding by growth-cone calcium transients. Nature.

[80] Song H.J., Ming G.L., Poo M.M. (1997). cAMP-induced switching in turning direction of nerve growth cones. Nature.

[81] Kater S.B., Davenport R.W., Guthrie P.B. (1994). Filopodia as detectors of environmental cues: Signal integration through changes in growth cone calcium levels. Prog. Brain Res..

[82] Sutherland D.J., Goodhill G.J. (2015). The interdependent roles of Ca^2+^ and cAMP in axon guidance. Dev. Neurobiol..

[83] Forbes E.M., Thompson A.W., Yuan J., Goodhill G.J. (2012). Calcium and cAMP levels interact to determine attraction versus repulsion in axon guidance. Neuron.

[84] Akiyama H., Fukuda T., Tojima T., Nikolaev V.O., Kamiguchi H. (2016). Cyclic Nucleotide Control of Microtubule Dynamics for Axon Guidance. J. Neurosci..

[85] Dickson B.J. (2002). Molecular mechanisms of axon guidance. Science.

[86] Johnson D.E., Williams L.T. (1993). Structural and functional diversity in the FGF receptor multigene family. Adv. Cancer Res..

[87] Raffioni S., Thomas D., Foehr E.D., Thompson L.M., Bradshaw R.A. (1999). Comparison of the intracellular signaling responses by three chimeric fibroblast growth factor receptors in PC12 cells. Proc. Natl. Acad. Sci. USA.

[88] Ota S., Tonou-Fujimori N., Yamasu K. (2009). The roles of the FGF signal in zebrafish embryos analyzed using constitutive activation and dominant-negative suppression of different FGF receptors. Mech. Dev..

[89] Itoh N., Ornitz D.M. (2008). Functional evolutionary history of the mouse Fgf gene family. Dev. Dyn..

[90] Jaye M., Schlessinger J., Dionne C.A. (1992). Fibroblast growth factor receptor tyrosine kinases: Molecular analysis and signal transduction. Biochim. Biophys. Acta.

[91] Bottcher R.T., Niehrs C. (2005). Fibroblast growth factor signaling during early vertebrate development. Endocr. Rev..

[92] Schlessinger J., Plotnikov A.N., Ibrahimi O.A., Eliseenkova A.V., Yeh B.K., Yayon A., Linhardt R.J., Mohammadi M. (2000). Crystal structure of a ternary FGF-FGFR-heparin complex reveals a dual role for heparin in FGFR binding and dimerization. Mol. Cell.

[93] Polanska U.M., Fernig D.G., Kinnunen T. (2009). Extracellular interactome of the FGF receptor-ligand system: Complexities and the relative simplicity of the worm. Dev. Dyn..

[94] Gonzalez A.M., Berry M., Maher P.A., Logan A., Baird A. (1995). A comprehensive analysis of the distribution of FGF-2 and FGFR1 in the rat brain. Brain Res..

[95] Tole S., Gutin G., Bhatnagar L., Remedios R., Hebert J.M. (2006). Development of midline cell types and commissural axon tracts requires Fgfr1 in the cerebrum. Dev. Biol..

[96] Cirillo A., Arruti C., Courtois Y., Jeanny J.C. (1990). Localization of basic fibroblast growth factor binding sites in the chick embryonic neural retina. Differentiation.

[97] Ornitz D.M., Xu J., Colvin J.S., McEwen D.G., MacArthur C.A., Coulier F., Gao G., Goldfarb M. (1996). Receptor specificity of the fibroblast growth factor family. J. Biol. Chem..

[98] Philippe J.M., Garces A., deLapeyiere O. (1998). Fgf-R3 is expressed in a subset of chicken spinal motorneurons. Mech. Dev..

[99] Thomson R.E., Kind P.C., Graham N.A., Etherson M.L., Kennedy J., Fernandes A.C., Marques C.S., Hevner R.F., Iwata T. (2009). Fgf receptor 3 activation promotes selective growth and expansion of occipitotemporal cortex. Neural Dev..

[100] Asai T., Wanaka A., Kato H., Masana Y., Seo M., Tohyama M. (1993). Differential expression of two members of FGF receptor gene family, FGFR-1 and FGFR-2 mRNA, in the adult rat central nervous system. Brain Res. Mol. Brain Res..

[101] Miyake A., Hattori Y., Ohta M., Itoh N. (1996). Rat oligodendrocytes and astrocytes preferentially express fibroblast growth factor receptor-2 and -3 mRNAs. J. Neurosci. Res..

[102] Zhang X., Ibrahimi O.A., Olsen S.K., Umemori H., Mohammadi M., Ornitz D.M. (2006). Receptor specificity of the fibroblast growth factor family. The complete mammalian FGF family. J. Biol. Chem..

[103] Fuhrmann V., Kinkl N., Leveillard T., Sahel J., Hicks D. (1999). Fibroblast growth factor receptor 4 (FGFR4) is expressed in adult rat and human retinal photoreceptors and neurons. J. Mol. Neurosci..

[104] Eswarakumar V.P., Lax I., Schlessinger J. (2005). Cellular signaling by fibroblast growth factor receptors. Cytokine Growth Factor Rev..

[105] Beenken A., Mohammadi M. (2009). The FGF family: Biology, pathophysiology and therapy. Nat. Rev. Drug Discov..

[106] Peters K.G., Marie J., Wilson E., Ives H.E., Escobedo J., Del Rosario M., Mirda D., Williams L.T. (1992). Point mutation of an FGF receptor abolishes phosphatidylinositol turnover and Ca^2+^ flux but not mitogenesis. Nature.

[107] Doherty P., Walsh F.S. (1996). CAM-FGF receptor interactions: A model for axonal growth. Mol. Cell. Neurosci..

[108] Jossin Y., Goffinet A.M. (2007). Reelin signals through phosphatidylinositol 3-kinase and Akt to control cortical development and through mTor to regulate dendritic growth. Mol. Cell. Biol..

[109] Konno D., Yoshimura S., Hori K., Maruoka H., Sobue K. (2005). Involvement of the phosphatidylinositol 3-kinase/rac1 and cdc42 pathways in radial migration of cortical neurons. J. Biol. Chem..

[110] Poulopoulos A., Murphy A.J., Ozkan A., Davis P., Hatch J., Kirchner R., Macklis J.D. (2019). Subcellular transcriptomes and proteomes of developing axon projections in the cerebral cortex. Nature.

[111] Yang J.J., Bertolesi G.E., Dueck S., Hehr C.L., McFarlane S. (2019). The Expression of Key Guidance Genes at a Forebrain Axon Turning Point Is Maintained by Distinct Fgfr Isoforms but a Common Downstream Signal Transduction Mechanism. eNeuro.

[112] Gallo G., Letourneau P.C. (1998). Localized sources of neurotrophins initiate axon collateral sprouting. J. Neurosci..

[113] Atwal J.K., Massie B., Miller F.D., Kaplan D.R. (2000). The TrkB-Shc site signals neuronal survival and local axon growth via MEK and P13-kinase. Neuron.

[114] Ming G., Song H., Berninger B., Inagaki N., Tessier-Lavigne M., Poo M. (1999). Phospholipase C-gamma and phosphoinositide 3-kinase mediate cytoplasmic signaling in nerve growth cone guidance. Neuron.

[115] Brewer J.R., Mazot P., Soriano P. (2016). Genetic insights into the mechanisms of Fgf signaling. Genes Dev..

[116] Reuss B., von Bohlen und Halbach O. (2003). Fibroblast growth factors and their receptors in the central nervous system. Cell Tissue Res..

[117] Dent E.W., Gertler F.B. (2003). Cytoskeletal dynamics and transport in growth cone motility and axon guidance. Neuron.

[118] Henley J., Poo M.M. (2004). Guiding neuronal growth cones using Ca^2+^ signals. Trends Cell Biol..

[119] Dent E.W., Kwiatkowski A.V., Mebane L.M., Philippar U., Barzik M., Rubinson D.A., Gupton S., Van Veen J.E., Furman C., Zhang J. (2007). Filopodia are required for cortical neurite initiation. Nat. Cell Biol..

[120] Tanaka E., Sabry J. (1995). Making the connection: Cytoskeletal rearrangements during growth cone guidance. Cell.

[121] Schaefer A.W., Kabir N., Forscher P. (2002). Filopodia and actin arcs guide the assembly and transport of two populations of microtubules with unique dynamic parameters in neuronal growth cones. J. Cell Biol..

